# Dysfunction in the coagulation system and schizophrenia

**DOI:** 10.1038/tp.2015.204

**Published:** 2016-01-05

**Authors:** S Hoirisch-Clapauch, O B Amaral, M A U Mezzasalma, R Panizzutti, A E Nardi

**Affiliations:** 1 Department of Hematology, Hospital Federal dos Servidores do Estado, Ministry of Health, Rio de Janeiro, Brazil; 2 Department of Medical Biochemistry, Medical Biochemistry Institute, Federal University of Rio de Janeiro, Rio de Janeiro, Brazil; 3 Institute of Psychiatry, Federal University of Rio de Janeiro, Rio de Janeiro, Brazil; 4 National Institute for Translational Medicine, Instituto Nacional de Ciência e Tecnologia - Translacional em Medicina, Rio de Janeiro, Brazil; 5 Basic-Clinical Neuroscience Program, Biomedical Sciences Institute, Federal University of Rio de Janeiro, Rio de Janeiro, Brazil

## Abstract

Although different hypotheses have been formulated to explain schizophrenia pathogenesis, the links between them are weak. The observation that five psychotic patients on chronic warfarin therapy for deep-vein thrombosis showed long-term remission of psychotic symptoms made us suspect that abnormalities in the coagulation pathway, specifically low tissue plasminogen activator (tPA) activity, could be one of the missing links. Our hypothesis is supported by a high prevalence of conditions affecting tPA activity in drug-naive schizophrenia, such as antiphospholipid antibodies, elevated cytokine levels, hyperinsulinemia and hyperhomocysteinemia. We recently screened a group of schizophrenia patients and controls for conditions affecting tPA activity. Free-protein S deficiency was highly prevalent among patients, but not found in controls. Free-protein S and functional protein C are natural anticoagulants that form complexes that inhibit tPA inhibitors. All participants had normal protein C levels, suggesting that protein S could have a role in schizophrenia, independent of protein C. Chronic patients and those studied during acute episodes had between three and six conditions affecting tPA and/or protein S activity, while patients in remission had up to two, which led us to postulate that multiple conditions affecting tPA and/or protein S activity could contribute to the full expression of schizophrenia phenotype. This paper describes the physiological roles of tPA and protein S, reviewing how their activity influences pathogenesis and comorbidity of schizophrenia. Next, it analyzes how activity of tPA and protein S is influenced by biochemical abnormalities found in schizophrenia. Last, it suggests future directions for research, such as studies on animal models and on therapeutic approaches for schizophrenia aiming at increasing tPA and protein S activity.

## Introduction

Schizophrenia has a substantial genetic basis, but environmental factors such as traumatic life events may increase the risk of psychotic symptoms.^1^ The disorder is characterized by impaired cognition, by hallucinations and delusions, referred to as positive symptoms, and by social isolation, flat affect and low motivation, known as negative symptoms.^1^

Antipsychotic medications usually attenuate positive symptoms, but fail to improve negative features or cognitive function.^2^ For example, 94% of 215 patients with schizophrenia spectrum disorder considered to be in remission had at least one residual symptom, mostly blunted affect, conceptual disorganization and social withdrawal.^3^ Given that residual symptoms can impair performance in school, affect performance at work, disrupt relationships and substantially affect quality of life, the need for more effective therapies is obvious.

Although different hypotheses have been proposed to explain schizophrenia pathogenesis, a link connecting them is missing. Finding the molecular link would allow for a better understanding of schizophrenia pathophysiology, which could lead to new therapeutic targets and better prognostic outcomes.

After noticing that five psychotic patients on chronic warfarin therapy for deep-vein thrombosis showed remission of psychotic symptoms, we assumed that defective modulation of the coagulation pathway might contribute to schizophrenia pathogenesis.^4^ In accordance with other studies, neuroimaging studies of our patients showed brain atrophy, but no ischemic lesions.^5, 6^

Searching for elements that modulate the coagulation pathway and also participate in processes that help prevent brain atrophy, only one candidate emerged: tissue plasminogen activator (tPA).

Conditions affecting the activity of tPA, such as hyperhomocysteinemia and antiphospholipid antibodies, have been consistently described in drug-naive schizophrenia.^7, 8, 9, 10^ We recently screened 70 drug-treated schizophrenia patients and 98 controls for these and other conditions affecting tPA activity.^11^ Persistent antiphospholipid antibodies were seen in 30% of the patients and none of the controls. Moreover, conditions that decrease tPA activity by increasing the activity of plasminogen activator inhibitor-1 (PAI-1, a major tPA inhibitor) were also highly prevalent among patients. Examples include the association of the 4G/5G polymorphism in the PAI-1 gene with hyperinsulinemia (20 vs 2%), hypertriglyceridemia (17 vs 5%) and hyperhomocysteinemia (24 vs 1%).

The same study detected a 22% prevalence of free-protein S deficiency in patients, while none of the controls presented the condition. As protein S is a cofactor of functional protein C, we were expecting to find a high prevalence of protein C deficiency among patients, but all participants had normal protein C levels. This and the 145-fold increased risk of having a first-degree relative with schizophrenia in patients with low free-protein S levels, compared with controls, led us to focus on protein S deficiency.^11^

Having observed that chronic schizophrenia patients and those studied during acute episodes had between three and six conditions, while patients in remission had up to two, we postulated that simultaneous conditions affecting tPA and/or protein S activity could contribute to the full expression of the schizophrenia phenotype.^11, 12^

In this review, we analyze the links between schizophrenia and elements that modulate the coagulation pathway, with particular emphasis on tPA and protein S. First, we describe the physiological roles of tPA and protein S, presenting evidence that the somatic comorbidity and laboratory abnormalities of schizophrenia can be related to decreased activity of tPA and/or protein S (Table 1). Next, we point out possible mechanisms by which low activity of tPA and/or protein S might contribute to schizophrenia pathogenesis. Finally, we suggest future directions for research, such as animal studies and therapeutic approaches based on normalization of tPA and protein S activity.

## tPA: mechanism of action in the blood and the brain

**Figure 1:**
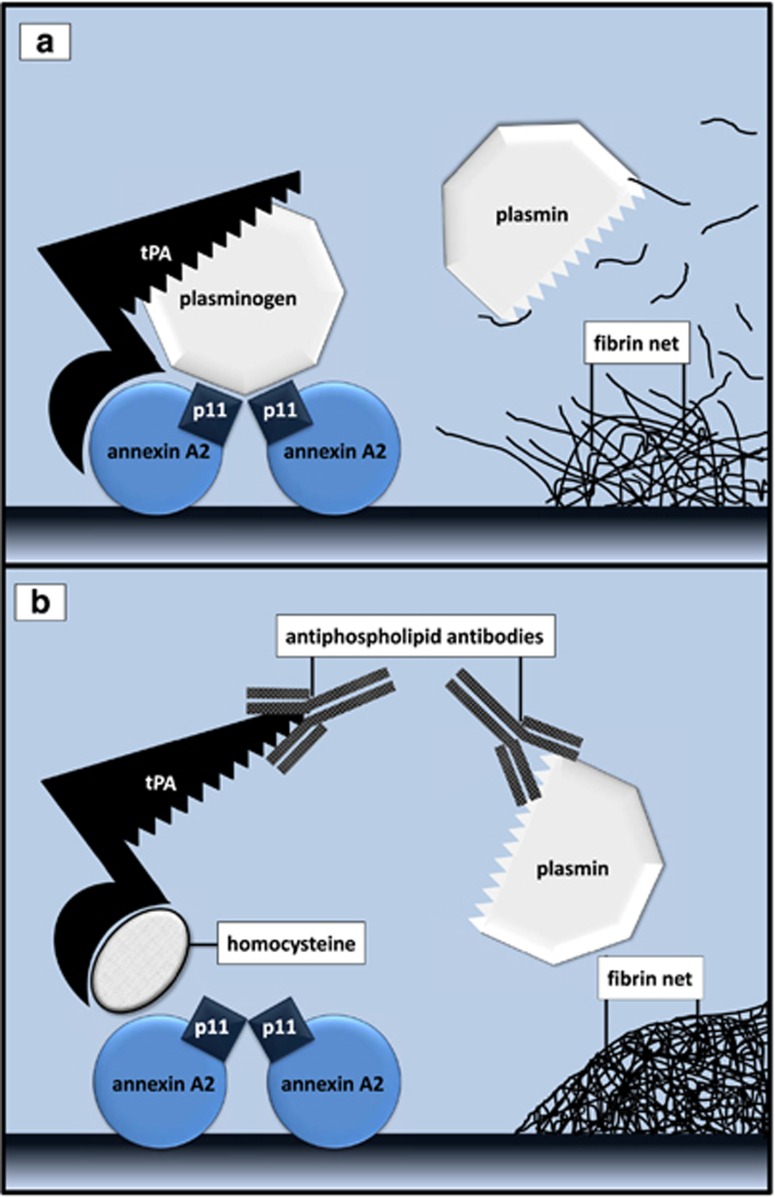
(**a**) Plasminogen and tPA must bind to the heterotetramer formed by two molecules of p11 and two molecules of annexin A2 to generate plasmin. Plasmin dissolves the fibrin net. (**b**). Antiphospholipid antibodies and homocysteine inhibit tPA-induced proteolysis and fibrinolysis. tPA, tissue plasminogen activator.

tPA mediates both protective and toxic mechanisms in the brain. Endothelial or recombinant tPA catalyzes the conversion of plasminogen to plasmin (Figure 1) and plasmin degrades fibrin clots, thus protecting against ischemic injury.^13^ On the other hand, an excessive amount of tPA may damage the blood–brain barrier, increasing the risk of edema and bleeding, both of which may exacerbate neuronal death.^14^

Neurons and glial cells also synthesize and release tPA, which is highly expressed in the cortex, amygdala, hippocampus and cerebellum.^13^ Following brain insults such as epileptic seizure, trauma or stroke, increased synthesis of tPA can overactivate excitatory receptors such as the N-methyl-D-aspartate (NMDA) receptor, which increases calcium permeability, causing neuronal damage and death.^14, 15^ While excessive NMDA receptor activation can damage neurons, the same occurs with its reduced activity.^14, 16^

Activation of NMDA receptors, reelin and neurotrophins, as well as dopamine release are some of the many neuroprotective mechanisms mediated by tPA or by plasmin that are defective in schizophrenia.^12, 14, 15, 16, 17, 18, 19, 20, 21^ The NMDA receptor stimulates neuronal migration and is involved in mechanisms of synaptic plasticity, a prerequisite for learning and memory skills. Proteolytic processing of reelin by tPA is fundamental for its function. Reelin stimulates dendrite and dendritic spine development and has an important role in learning and memory.^17^

Cleavage of proneurotrophins, such as of pro-brain-derived neurotrophic factor (pro-BDNF), is also mediated by tPA. While proneurotrophins induce dendritic and synaptic deterioration of cultured neurons, and even neuronal apoptosis, mature neurotrophins have opposite functions, promoting growth and remodeling of axons and dendrites, synaptic formation, function and plasticity, neuronal proliferation and survival.^18^ In the limbic system, tPA regulates the release of dopamine, a neurotransmitter involved in reward-mediated learning and memory.^19^

Studies in animal models suggest that tPA plays a critical role in the formation of various forms of synaptic plasticity and cognition. For example, in open field object exploration task, tPA-knockout mice express deficits in habituation and reactivity to spatial change, decreased rearing and poor initial object exploration, consistent with altered hippocampal and striatal function.^22^ In rats, subacute intranasal tPA treatment, initiated 7 days after traumatic brain injury, enhances neurogenesis in the dentate gyrus. In addition, intranasal tPA increases axonal sprouting of the corticospinal tract originating from the contralesional cortex into the denervated side of the cervical gray matter, and mature BDNF levels. As a result, treatment significantly improves cognitive and sensorimotor functional recovery.^23^

## tPA activity inhibition

In the brain, tPA is inhibited by PAI-1, PAI-2 and neuroserpin. PAI-1 is synthesized by arterial smooth muscle, fat cells, stroma cells of fat tissue and by hepatocytes, especially steatotic ones.^24^ Although PAI-1 immunoreactivity has been observed in human neurons and some reactive astrocytes,^24^ it has not been determined if these cells are able to synthesize PAI-1. PAI-2 is synthesized by keratinocytes, peritoneal macrophages, trophoblasts and microglia.^25^

**Figure 2:**
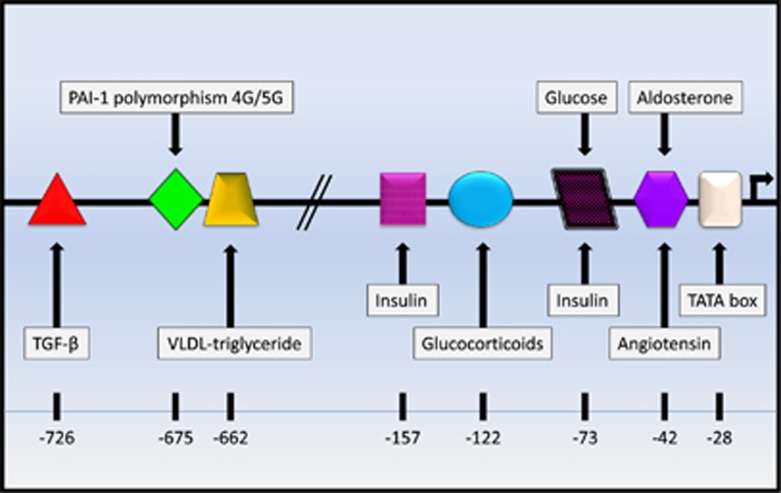
Schematic representation of PAI-1 promoter, showing the 4G/5G single nucleotide polymorphism (rs1799889) and enhancer elements. TATA box, the site of transcription initiation; TGF-β, transforming growth factor-β VLDL, very-low-density lipoprotein.

The PAI-1 promoter is activated by insulin, glucose, triglycerides, angiotensin (Figure 2) and leptin, a hormone produced by adipocytes.^26, 27^ A meta-analysis has estimated that >20% of the first-episode or unmedicated schizophrenia patients are obese or overweight, with a high prevalence of elevated fasting glucose and insulin levels, hypertriglyceridemia and high blood pressure.^28^ High levels of tumor necrosis factor-α, as seen in inflammatory disorders such as obesity, participate in the mechanism of insulin resistance in peripheral tissues.^29^ Pancreatic β cells compensate for insulin resistance by increasing insulin production.

Neuroserpin co-localizes with the secretory protein chromogranin B in large dense core vesicles. Chromogranins seem to play an on/off switch role for secretory granule biogenesis.^30^ An association between chromogranin B polymorphisms and schizophrenia has been reported in genome wide-association studies of the Chinese Han and Japanese populations. Although it is known that the polymorphism results in reduced levels of chromogranin B,^31^ the relationship between chromogranin and tPA in schizophrenia has not been elucidated yet.

Polymorphisms that reduce tPA activity have been also identified in schizophrenia. In a large sample of Japanese schizophrenia individuals, the association between the mental disorder and two single nucleotide polymorphisms of PLAT, the human tPA gene, rs2020922 and rs8178817, was highly significant.^32^

## Protein S: mechanism of action in the blood and the brain

**Figure 3:**
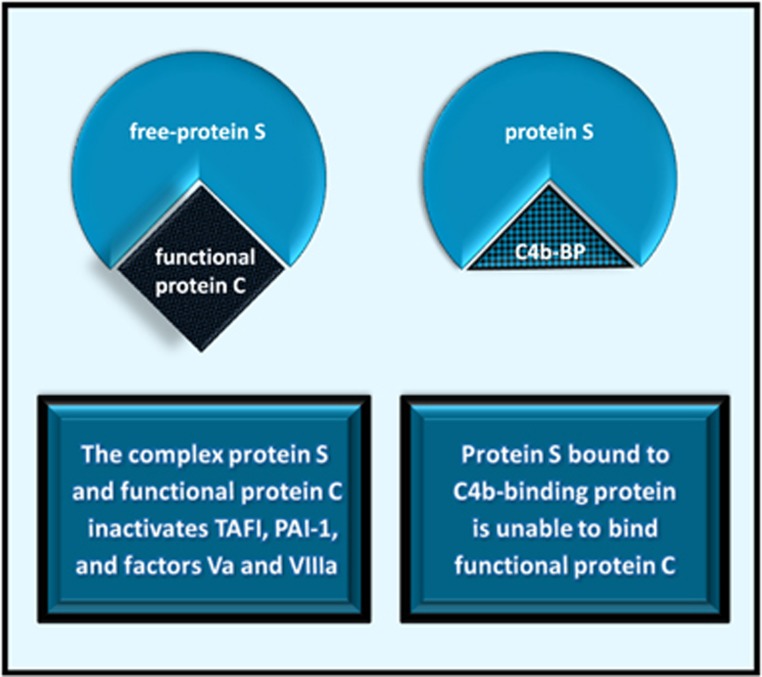
C4b-BP, C4b-binding protein; PAI-1, plasminogen activator inhibitor-1; TAFI, thrombin activatable fibrinolysis inhibitor.

Protein S is a natural anticoagulant that circulates in an active free form, or bound to C4b-binding protein, one of the complement inactivator proteins (Figure 3).^33^ Free-protein S forms a complex with activated protein C that inactivates factors Va and VIIIa, and two molecules that inhibit tPA: thrombin activatable fibrinolysis inhibitor and PAI-1.^33, 34^ Anticoagulant properties of protein S that do not depend on protein C include factor Xa and prothrombin inhibition.^35^

Roles for protein S beyond coagulation include neuroprotective and anti-inflammatory effects, which have been demonstrated in a murine *in vivo* model of ischemic stroke.^36^ A direct neuronal protective effect has been also shown in cultured cortical neurons challenged with hypoxia and aglycemia, followed by reoxygenation.^37^ Although it has previously been assumed that the phagocytosis of neurons is always preceded by their commitment to cell death, there is evidence that phagocytosis can mediate the death of viable neurons during development, inflammation and neuropathology.^38^ Protein S supports survival, proliferation and differentiation of viable neurons and neuronal stem cells via a mechanism that involves Tyro3, Axt and Mer (TAM) receptors.^37, 39^ TAM receptors, expressed by both astrocytes and microglia, are tyrosine kinase receptors that regulate cell proliferation and survival, cell adhesion and migration. As a TAM receptor ligand, protein S increases the levels of protein Bcl-2, an apoptotic suppressor in a variety of cells, including neurons.^39^

## Protein S inhibition

Protein S plasma levels are determined by genetic and environmental factors, including sex, hormonal status, smoking, age and disease.^35^ Inflammatory disorders are usually accompanied by decreased protein S activity, due to increased levels of C4b-binding protein. Hereditary deficiency of protein S deficiency is unlikely to be detected by genome wide-association studies because almost 200 mutations have been characterized in the PROS1 gene.^35^

## Links between clinical comorbidity of schizophrenia and low activity of tPA and/or protein S

Inherited and acquired conditions decreasing activity of tPA and/or protein S may affect clot lysis and extracellular matrix proteolysis, both of which may increase the risk of cardiovascular disease, thrombotic events and pregnancy complications. By affecting extracellular matrix proteolysis, low tPA activity may also contribute to a lower-than-expected risk of cancer.

### Cardiovascular risk and thrombotic events

Meta-analyses have consistently shown that ischemic cardiovascular disease reduces the life expectancy of schizophrenia patients, which is about 15 years less than that of the general population. Besides, more than two-thirds of the patients with schizophrenia die of coronary heart disease, compared with approximately half of the general population.^40, 41^ Increased cardiovascular risk has been ascribed to antipsychotic medication side effects, to cigarette smoking, to sedentary behavior and to immobility, such as in physical restraint or stupor.^42^

Low activity of tPA and/or protein S may also increase the risk of cardiovascular events. This is because a myocardial infarction may occur when an atherosclerotic plaque rupture triggers the formation of a blood clot that reduces blood flow through the coronary arteries. PAI-1, the major inhibitor of tPA, blocks the formation of plasmin, thereby preventing clot dissolution.^43^ Individuals with unstable angina and myocardial infarction usually have increased PAI-1 levels and activity.^43^

Schizophrenia patients seem to be at increased risk of thromboembolic events. By impairing anticoagulation and fibrinolysis, low activity of tPA and/or protein S may contribute to the problem.

### Pregnancy complications

Several studies have demonstrated that mothers with psychotic disorders are less likely to receive antenatal care and are at a higher risk of substance, alcohol and tobacco abuse than controls. However, even after controlling for these risk factors, the diagnosis of maternal schizophrenia remains predictive of adverse obstetric outcomes, such as intrauterine growth restriction, stillbirth and prematurity.^44, 45^

Low tPA activity may possibly contribute to the adverse outcomes. A healthy pregnancy depends on embryo implantation, trophoblast invasion, placental angiogenesis and placental vessel remodeling, a process that accompanies exponential fetal growth.^45^ tPA and/or plasmin participate in all these processes.^46^ Hereditary protein S deficiency, increasing the risk of placental vessel thrombosis, may also increase the risk of intrauterine growth restriction and preterm delivery.^47^

### Lower-than-expected risk of cancer

There seems to be a discrepancy between cancer incidence and exposure to cancer risk factors in schizophrenia, consistent with a protective effect.^48,49^ The lower-than-expected risk of lung cancer is particularly impressive, considering the high prevalence of heavy smokers among schizophrenia individuals. Overall risk of neoplastic disorders is also significantly reduced among unaffected parents and siblings of patients.^50, 51^

Tumor cells have the ability to penetrate the extracellular matrix, and, in the case of metastasis, the blood vessels. The degradation of surrounding tissues, a crucial step in tumor cell invasion and metastasis formation,^52, 53^ involves tPA and plasmin. In addition, proteolytic processing of extracellular matrix components by plasmin or tPA releases vascular endothelial growth factor and other molecules that regulate tumor angiogenesis.^52, 53^

## Links between biochemical abnormalities of schizophrenia and low tPA and/or protein S activity

Biochemical abnormalities commonly found in schizophrenia which may impair tPA and/or protein S activity include hypercortisolemia, increased cytokine levels, hyperhomocysteinemia and antiphospholipid antibodies, such as lupus anticoagulant and IgG and IgM anticardiolipin antibodies.

### Hypercortisolemia

Individuals with first-episode psychosis are more likely to have experienced traumatic events than the general population.^54^ Cortisol release from the adrenal glands increases several fold after exposure to a stressor.^55^

First-episode patients, treated with antipsychotics for <2 weeks, show significantly higher cortisol levels than controls.^54^ PAI-1 promoter responds to cortisol (Figure 2).^26^ Another mechanism by which cortisol decreases tPA levels involves glutamate. Glucocorticoids rapidly induce glutamate release in the hippocampus.^55^ Extracellular glutamate inhibits release of tPA by brain cells responsible for its recycling: astrocytes.^56^

The hypothesis that glucocorticoids have a role in schizophrenia pathogenesis is supported by the fact that patients taking glucocorticoids and those with Cushing's syndrome may develop psychotic symptoms.^57^ Moreover, it has been shown that subjects at high risk of schizophrenia who progress to the disorder have higher baseline cortisol levels than those who do not progress to psychosis or controls.^58^

### Elevated cytokine levels

Acute psychotic episodes are usually characterized by increased levels of cytokines such as interleukin (IL)-1β, IL-6 and transforming growth factor (TGF)-β.^59^ Cytokines are key regulators of inflammation that activate and recruit immune cells, increase blood supply and enhance vascular permeability.^60^ Elevated cytokine levels are commonly found in schizophrenia,^59^ accompanying obesity, periodontitis or other inflammatory disorders.

Components of the inflammatory response such as TGF-β may stimulate the synthesis of PAI-1 (Figure 2).^26^ Inflammation also decreases protein S activity, either by increasing C4b-binding protein levels (Figure 3) or by facilitating the proteolytic inactivation of protein S, possibly through neutrophil proteases.^61^ Conditions characterized by low protein S activity, such as systemic lupus erythematosus, Behçet's disease and Sjögren's syndrome may present with cognitive impairment, delusions and hallucinations.^9, 62^ In addition, puerperal psychosis occurs when protein S levels are low, regardless of systemic inflammation.^63^

### Elevated homocysteine levels

**Figure 4:**
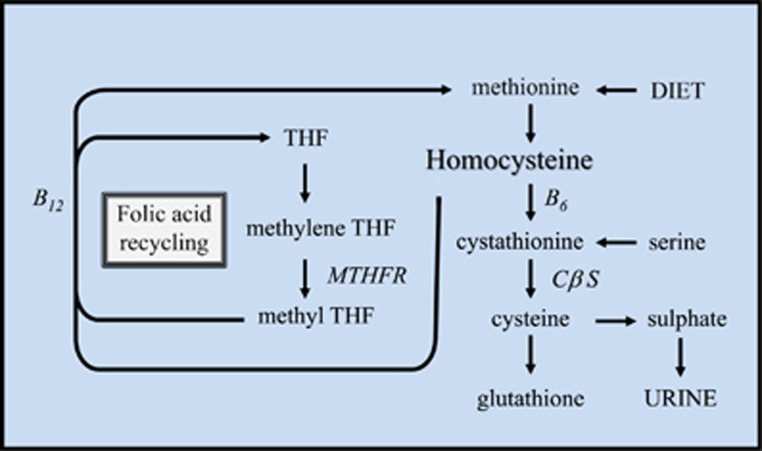
Homocysteine may be recycled to methionine or eliminated in urine as sulfate. Under conditions in which excess methionine is present, homocysteine condenses with serine to form cystathionine in a reaction catalyzed by the vitamin B6–dependent rate-limiting enzyme cystathionine β synthase. B12, vitamin B12; B6, vitamin B6; CβS, cystathionine β-synthase; MTHFR, methylene-tetrahydrofolate reductase; THF, tetrahydrofolate. Adapted from ref. 66.

Homocysteine is not a dietary constituent: it is formed upon demethylation of methionine. Homocysteine may be remethylated to methionine by N5-methyltetrahydrofolate in the presence of vitamin B12 or converted to cysteine by cystathionine-β-synthase, a vitamin B6-containing enzyme (Figure 4).^64^ Homocysteine plasma levels correlate with homocysteine brain levels^64^ and both folate deficiency and hyperhomocysteinemia are prevalent among first-episode, drug-naive schizophrenia patients.^65^

Different explanations for the association between elevated homocysteine levels and schizophrenia have been provided. Folate deficiency may induce neurodegeneration by increasing reactive oxygen species production and cytosolic calcium accumulation.^65^ Besides, homocysteine may damage neuronal DNA, triggering apoptosis.^64^ A third explanation fits into this model: homocysteine prevents tPA binding to annexin A2, which affects catalytic properties of tPA (Figure 1).

### Elevated levels of antiphospholipid antibodies

Antiphospholipid antibodies such as anticardiolipin antibodies and lupus anticoagulant are highly prevalent in drug-naive schizophrenia.^9, 10, 11^ The diagnosis of antiphospholipid antibody syndrome requires the persistence of antibodies, plus either a thrombotic event or an obstetric complication due to abnormal placentation.^67^ Patients with antiphospholipid antibodies may present with cognitive dysfunction or full-blown psychosis, independent of brain ischemia.^4, 8^

Based on the finding that antiphospholipid antibodies may increase blood–brain barrier permeability, it was postulated that the deleterious effects of these antibodies in the central nervous system were dependent on their binding to neurons, glia cells, oligodendrocytes and microglia.^9^

It is also possible that the link between antiphospholipid antibodies and mental disorders involves their inhibition of tPA and/or protein S activity. Antiphospholipid antibodies may directly inhibit tPA, plasminogen, plasmin or proteins that participate in the process of tPA and plasminogen assembling, such as β2 glycoprotein-1 and annexin A2 (Figure 1).^68^ Antiphospholipid antibodies against protein S have been also described.^69^

## Links between schizophrenia hypotheses and low activity of tPA and/or protein S

Low activity of tPA and/or protein S might link different hypotheses for schizophrenia, including the neuropil hypothesis, the NMDA receptor hypothesis, the dopaminergic hypothesis and the hypothesis correlating an adverse fetal environment with an increased risk of developing schizophrenia.

### Neuropil hypothesis

In schizophrenia, brain atrophy reflects loss of neuropil—the network of dense synaptic contacts formed by unmyelinated axons, dendrites and glial processes, rather than neuronal loss.^70^ Neuropil atrophy, observed mostly in the prefrontal region, hippocampus and thalamus,^5^ is thought to underlie changes in synaptic, dendritic and axonal organization that have been correlated to cognitive dysfunction.^6^ Decreased neurotrophin availability and reduced reelin expression are important contributors to brain atrophy in schizophrenia.^6^ The neuropil hypothesis supports the involvement of tPA in the pathogenesis of schizophrenia as tPA mediates proteolytic processing that activates neurotrophins and reelin.

### Reduced activity of NMDA receptor

Evidence indicates that hypofunction of the NMDA receptor, impairing synaptic plasticity, may contribute to the pathophysiology of schizophrenia. tPA may also have a role in the NMDA receptor hypothesis, because proteolytic activity of tPA and plasmin enhances NMDA receptor signaling.^15, 16^

### Dopaminergic hypothesis

Positron emission tomography and single-photon emission computed tomography have shown that impairment of dopaminergic transmission in schizophrenia is mostly pre-synaptic, and affects dopamine synthesis capacity, baseline synaptic dopamine levels and dopamine release.^71^ Dopamine-activated post-synaptic neurons release tPA into the extracellular space. tPA converts plasminogen to plasmin, and plasmin acts on pre-synaptic dopaminergic neurons via plasminogen activator receptor-1 to potentiate the activity-dependent release of dopamine in the nucleus accumbens.^72^

### Adverse fetal environment

Individuals born prematurely or with low-birth weight are at increased risk for developing schizophrenia.^73^ Assuming a high prevalence of hereditary protein S deficiency or inherited conditions reducing tPA activity among mothers of schizophrenia patients, one would expect in this group an increased prevalence of pregnancy complications related to abnormal placentation. In this setting, a preterm delivery or a small-for-gestational-age offspring would be a parallel occurrence, not an element belonging to schizophrenia pathogenesis.

## Recommendations for future research

### Protein S deficiency

We recommend that free-protein S levels be assessed in a large series of first-episode drug-naive patients with schizophrenia and their first-degree relatives. The prevalence of psychosis in thrombophilia patients with hereditary protein S deficiency also remains to be determined.

### Risk of thromboembolic events

A large multicentric study is needed to compare the prevalence of thrombotic events before the diagnosis of schizophrenia with that of the general population. The Computerized Registry of Patients with Venous Thromboembolism (RIETE, www.riete.org) is currently looking for a possible association between mental disorders and thrombotic tendency.

### Autoimmune disorders

We recommend that tPA activity and free-protein S levels be assessed in patients with autoimmune disorders with and without psychosis, especially those with lupus erythematosus or antiphospholipid antibody syndrome.

### Interventions aiming at increasing tPA or protein S activity

Controlled studies are required to determine how interventions aiming specifically at increasing tPA and protein S activity affect the course of schizophrenia. It should be highlighted that because restoration of hippocampal and prefrontal cortex circuitry in schizophrenia patients requires multiple sequential steps, short-term improvement after normalization of tPA and protein S activity is not expected to occur. Interventions effective in increasing tPA levels include lifestyle modifications, vitamin supplementation aiming at normalizing homocysteine levels, treatment of inflammation, anticoagulants and electroconvulsive therapy. Intervention studies should stratify patients as users of anticoagulants that inhibit protein S synthesis, such as warfarin or acenocoumarol, and users of other anticoagulants. Although promising, intranasal tPA has not been tested in humans.

Evidence indicates that lifestyle interventions, such as exercise,^74, 75^ a low-carbohydrate diet^76^ and diets for weight loss,^77^ can alleviate psychotic symptoms. It has not been defined whether higher-calorie-expenditure exercises (45 to 60 min per session, almost daily walking) are more effective than short distance walking (25 to 40 min, few times per week, or multiple exercise modalities) in alleviating psychotic symptoms.^78^ We suggest that protein supplementation be tested in controlled studies, as a strategy to correct hyperinsulinemia and restoring tPA activity.

Many physicians will be reluctant to conduct studies with anticoagulant medications in therapeutic doses, due to the risk of bleeding. Patients with a previous thrombotic event and persistent antiphospholipid antibodies have a high risk of recurrence and most authorities agree that they should be placed on long-term anticoagulation.^67^ Schizophrenia patients with such profile are thus ideal candidates for preliminary studies. Although it is known that tPA crosses the intact blood–brain barrier,^79^ whether warfarin increases tPA synthesis in the central nervous system remains to be defined.

Electroconvulsive therapy may alleviate psychotic symptoms. In rats, tPA activity rapidly increases by over 50% after electroconvulsive shock, and remains elevated for more than 24 h.^80^ tPA mediates a variety of chemical reactions underlying the mechanism of action of electroconvulsive therapy.^81^ We recommend that patients refractory to the procedure be screened for conditions affecting tPA activity, such as hyperhomocysteinemia or antiphospholipid antibodies (Figure 1).

### Animal studies

Animal studies are very suitable to generate evidence of a direct causal relationship between tPA or protein S and schizophrenia-like symptoms. More careful examination of the tPA-knockout mice should be performed, focusing on schizophrenia-related findings and behaviors such as social cognition. Another strategy would be to study the developmental and behavioral effects of transient and/or conditional tPA gene knockdown. Protein S knockout mice are not viable,^82^ but protein S-deficient mice can reach adulthood. Studies of brain development and behavior should be performed in protein S-deficient mice, as well as in animals with transient and/or conditional protein S gene knockdown. The reversal of developmental and behavioral findings in those animal models with antipsychotic drugs and/or warfarin should also be investigated.

We also recommended that tPA levels, tPA activity, and total- and free-protein S levels be evaluated in animal models of schizophrenia with good etiologic, phenotypic and predictive validity. Assuming that low tPA activity will be detected in these animals, it would be interesting to assess if warfarin therapy can change behavioral phenotypes with or without metabolic interventions aiming at preventing hyperinsulinemia and hypertriglyceridemia. It would be also important to assess if intra-cerebral levels of tPA and protein S correlate with their peripheral levels. It is well-known that cortisol, insulin and triglycerides increase PAI-1 plasma levels (Figure 2),^26^ but their effect on neuronal PAI-1 levels has not been reported.

It has been reported that tPA-null mice are resistant to neuronal destruction after intra-hippocampal injections of excitotoxins,^83^ but it is unknown whether these animals are more vulnerable to stress-induced neuronal damage.

## Conclusion

Future research is needed to elucidate the exact role of tPA and protein S in the pathogenesis of schizophrenia and to determine the impact of interventions aiming specifically at correcting activity of tPA and protein S on the course of schizophrenia.

## Figures and Tables

**Table 1:** Influence of low activity of tPA and/or protein S on schizophrenia features

*Low activity of tPA and/or protein S*	
*Clinical comorbidity of schizophrenia*	*Biochemical features of schizophrenia*
Cardiovascular risk	Hypercortisolemia
Thrombotic tendency	Elevated cytokine levels
Pregnancy complications	Hyperhomocysteinemia
Lower-than-expected risk of cancer	Hyperinsulinemia
*Schizophrenia pathogenesis*	Hypertriglyceridemia
Reduced neutrophil	Antiphospholipid antibodies (lupus
Abnormal NMDA receptor activation	anticoagulant and anticardiolipin antibodies)
Dopaminergic hypothesis	Low free-protein S levels?
Adverse fetal environment	

Abbreviations: NMDA, N-methyl-D-aspartate; tPA, tissue plasminogen activator.
